# Effects of cerebrovascular disease on amyloid precursor protein metabolites in cerebrospinal fluid

**DOI:** 10.1186/1743-8454-7-10

**Published:** 2010-07-30

**Authors:** Per Selnes, Kaj Blennow, Henrik Zetterberg, Ramune Grambaite, Lars Rosengren, Lisbeth Johnsen, Vidar Stenset, Tormod Fladby

**Affiliations:** 1 Department of Neurology, Akershus University Hospital, Norway; 2 Faculty Division Akershus University Hospital, University of Oslo, Norway; 3 Institute of Neuroscience and Physiology, Department of Psychiatry and Neurochemistry, the Sahlgrenska Academy at University of Gothenburg, Sweden; 4 Institute of Neuroscience and Physiology, Department of Neurology, the Sahlgrenska Academy at University of Gothenburg, Sweden; 5 Department of Neurosurgery, Oslo University Hospital Ullevål, Norway; 6 Department of Psychology, University of Oslo, Norway

## Abstract

**Background:**

Alzheimer's disease (AD) and cerebrovascular disease (CVD) including chronic small vessel disease of the brain (SVD) are the most frequent causes of dementia. AD is associated with metabolism of amyloid precursor protein (APP) and low levels of amyloid-β peptide (Aβ) X-42 in the cerebrospinal fluid (CSF). CVD and SVD are established risk factors for AD, brain white matter lesions (WML) are established surrogate markers for SVD and are also associated with reduced CSF AβX-42.

A cohort survey was performed to examine whether SVD or acute CVD affects APP metabolism and to explore a potential association between WML and APP metabolism in two groups; cognitively impaired patients, subjective and mild (SCI and MCI) and stroke patients. Through measurements of CSF APP metabolite levels in patients with a wide range of WML volumes, this study aimed to determine how SVD influences APP metabolism.

**Methods:**

Sixty-three patients were included: 37 with subjective cognitive impairment (SCI) or mild cognitive impairment (MCI) without stroke, and 26 after acute stroke. Chronic and acute WML volume and infarct volume were determined by magnetic resonance imaging (MRI) post-scan processing, and CSF levels of α- and β-cleaved soluble APP (sAPP-α and sAPP-β, AβX-38, AβX-40 and AβX-42) were determined. The Mann-Whitney test was used to compare the patient groups. Chronic and acute WML volumes, infarct volume, age, and sex were used as predictors for CSF biomarker levels in linear regression analysis.

**Results:**

CSF levels of sAPP-α and sAPP-β were strongly correlated (*r *= 0.95, *p *< 0.001) and lower levels of these biomarkers were found in the stroke group than in the SCI/MCI group; median sAPP-α 499.5 vs. 698.0 ng/mL (*p *< 0.001), sAPP-β 258.0 vs. 329.0 ng/mL (*p *< 0.005). CSF levels of sAPP-α, sAPP-β, AβX-38, AβX-40 and AβX-42 were inversely correlated with chronic WML volume (*p *≤ 0.005; *p *≤ 0.01; *p *≤ 0.01; *p *≤ 0.05; *p *≤ 0.05 respectively), but not with acute WML or infarct volumes.

**Conclusions:**

Lower CSF levels of sAPP-α and sAPP-β in the stroke group than in the SCI/MCI group and an inverse correlation with chronic WML indicate that ischemia lowers the levels of CSF sAPP metabolites and suggests that APP axonal transport or metabolism may be affected in SVD of the brain.

## Background

Alzheimer's disease (AD) and cerebrovascular disease (CVD) are the most frequent causes of dementia. Familial AD is associated with metabolism of the transmembrane amyloid precursor protein (APP) and mutations in the APP gene [1,2], while less is known about the etiology of sporadic AD [3]. However, findings in histopathology [4] and molecular imaging [5] imply that amyloid metabolism is also involved in sporadic cases. After fast axonal transport of APP to synaptic terminals [6], α- or β-secretase cleaves the protein into soluble APP (sAPP-α or sAPP-β) and C-terminal fragments (αCTFs and βCTFs) [7]. Subsequent cleavage of βCTFs (by γ-secretase) yields amyloid β (Aβ) peptides X-38, X-40 and X-42 [8]. AβX-42 is prone to deposition in amyloid plaques [9], and an association between low levels of AβX-42 in cerebrospinal fluid (CSF) and presence of amyloid plaques has been shown both in molecular imaging [5] and post-mortem histopathological studies [10]. Low CSF AβX-42 is also a predictor of AD [11]. APP and sAPP-α are important factors for neurite outgrowth [12] and neuronal plasticity and memory [13,14].

We have found similar CSF levels of sAPP-α and sAPP-β in sporadic AD and mild cognitive impairment (MCI) [15] to control subjects [16]. Subjective cognitive impairment (SCI) [17] is a pre-MCI stage characterized by subjectively impaired cognition which is not demonstrable with objective screening tests. SCI [18], MCI, white matter lesions (WML) [19] and stroke [20,21] all increase the risk of dementia and AD. WML are established surrogate markers of chronic small vessel disease of the brain (SVD) [22,23] and are frequently seen on T2-weighted magnetic resonance imaging (MRI) scans of individuals with and without dementia [24], and are present in increased amount in AD [25]. They are associated with reduced CSF levels of AβX-42 in various diseases [26-28], and an inverse correlation between WML volume and CSF level of sAPP-α and sAPP-β has been demonstrated in non-demented elderly people [29]. Experimental stroke [30] and ischemia [31] lead to an increased production of APP, upregulation of β-secretase activity [32], and an accumulation of Aβ peptides and APP around ischemic WMLs [31]. Axonal transport is impeded both by WML and APP metabolites [33,34]. Thus, APP over-expression may then impede axonal transport, also of APP, and impair neuronal plasticity and survival [35]. In ischemic conditions, CSF levels of APP metabolites may be influenced by increased gene expression, impeded axonal transport and deposition in plaques (which mainly contributes to reduced levels of CSF AβX-42) [10]. In the case of impeded transport, low cortical levels of APP [14] and sAPP-α [13] may also contribute to cognitive decline.

In this study, a hypothetical association between volume of WML and CSF concentration of sAPP metabolites (sAPP-α and sAPP-β) in cognitively impaired patients was tested by comparing CSF levels of these metabolites in patients with SCI or MCI to those in post-stroke patients and to MRI-based quantitative measures of brain ischemia (WML) in both patient groups. Through measurement of APP metabolite levels in patients with a wide range of WML volumes, this study aimed to determine how SVD influences APP metabolism.

## Methods

Patients with SCI and MCI were recruited from a university-hospital based memory clinic between September 2005 and December 2007. Inclusion criteria were age 40-79, established SCI or MCI for at least 6 months, Global Deterioration Scale [36] score 2 or 3 (scores 4 and higher are per definition dementia) as determined from a clinical interview, Clinical Dementia Rating [37] ≤ 0.5 and results of screening tests performed at time of inclusion (mini-mental state examination MMSE [38]; Stepwise comparative status analysis parameters 13-20 [39]; fluency, interference and numeral-letter items from the I-flex [40]; and Cognistat [41]). Exclusion criteria were impaired activities of daily living, established psychiatric disorder, cancer, drug abuse, solvent exposure or anoxic brain damage. Thirty-seven patients fulfilled all criteria, and successfully underwent MRI and lumbar puncture.

Stroke patients were recruited from a university-hospital based stroke unit during 2007. Inclusion criteria for these patients were: cortical and lacunar supratentorial infarctions, classified by MRI, between 40 and 79 years of age, and cognitive complaints but MMSE score >23, no severe problems of language and visual/auditory neglect. Exclusion criteria were a history of medical or psychiatric disorder including depression. Twenty-six patients fulfilled all criteria, and successfully underwent MRI and lumbar puncture. Table 1 presents sex, age and MMSE scores for each patient group. All patients gave their written consent, and the regional ethics committee approved the study.

**Table 1:** Patient characteristics

Variable		Stroke	SCI/MCI
Sex	Men (total)	20 (26)	20 (37)

Age	Median	66.0	60.4
	
	Range	42-78	43-77

MMSE	Median	29.0	28.0
	
	Range	23-30	23-30

SCI: subjective cognitive impairment, MCI: mild cognitive impairment, MMSE: mini-mental state examination

### MRI

MRI scans were from two sites (site 1: 10 patients, all SCI/MCI; site 2: 53 patients). Site 1: Siemens Symphony 1.5 T (Siemens, Erlangen, Germany) with a conventional quadrature head coil. Two 3 D magnetization-prepared gradient echo (MP-RAGE), T1-weighted sequences in succession (TR/TE/TI/FA = 2730 ms/3.19 ms/1100 ms/15◦, matrix = 256 × 192), 128 sagittal slices, thickness = 1.33 mm, in-plane resolution of 1.0 × 1.33 mm. Site 2: Siemens Espree 1.5 T using two 3 D MP-RAGE, T1-weighted sequences in succession (TR/TE/TI/FA = 2400/3.65/1000/8◦, matrix = 240 × 192), 160 sagittal slices, thickness = 1.2 mm, in-plane resolution of 1.0 mm × 1.2 mm.

**Figure 1:**
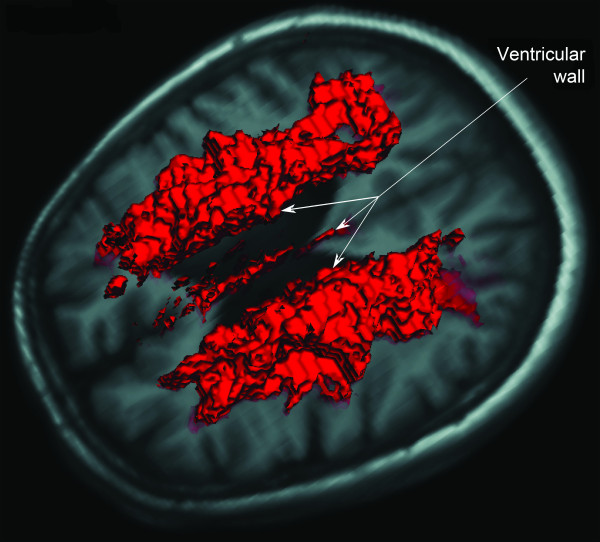
**Visualization of chronic white matter lesions obtained by MRI**. The hyper-intense areas in the FLAIR images (in red) are overlain onto the T1 images and shown in three dimensions. The ventricular walls (arrows), adjacent periventricular and subcortical hyper-intensities are visualized.

For the SCI/MCI patients MRI was performed after inclusion, whereas it was performed 3 months after the stroke for stroke patients. Acute stroke localization was determined during the acute stage hospitalization. At this stage, diffusion-weighted MRI was also performed if infarct localization was not evident from cerebral computed tomography and clinical examination. WML, including white matter hyperintensities associated with current or previous infarction(s), were quantified with a semi-automated method in the Nordic ICE clinical image processing and analysis software application (NordicNeuroLab AS, Norway). In the T2-weighted fluid attenuated inversion recovery (FLAIR) images, pixel values in white matter higher than two standard deviations (SD) above mean pixel value were defined as WML (Fig 1). White matter hyperintensities associated with current or previous infarctions were classified as acute or chronic WML. The former was also considered likely to represent acute post-stroke changes, possibly stroke penumbra volume and subtracted from total WML volume in post-stroke patients to determine the volume of chronic WML used for further analysis.

Using the FLAIR-weighted images, hypointensities classified as current or previous infarctions were quantified by manually assigning a freehand region of interest to the area. The areas of each slice were added and multiplied with slice thickness to obtain total volumes. Images from one MCI patient were excluded due to motion artifacts.

### CSF

CSF samples were collected by lumbar puncture through the L3/L4 or L4/L5 intervertebral-space. The lumbar puncture was performed consecutively after inclusion in the SCI/MCI group or, in the stroke group (7-10 days post stroke). CSF was collected in a polypropylene tube and centrifuged at 2,000 × *g *at +4°C for 10 min. The supernatant was removed, gently mixed to avoid possible gradient effects, and stored within one hour at -80°C, pending biochemical analyses.

CSF concentrations of sAPP-α and sAPP-β, and AβX-38, AβX-40 and AβX-42 were determined using the MSD^® ^sAPP-α/sAPP-β Multiplex Assay and MSD^® ^Aβ Triplex Assay as described by the manufacturer (Meso Scale Discovery, Gaithersburg, MD, USA). Coefficients of variation were < 10% for all analyses. All analyses from the MCI and stroke groups were performed in the same batch.

### Statistics

The statistics software package PASW 18 (SPSS Inc, USA) was used for statistical analysis. Linear regression was used to regress out age and sex, providing standardized residuals for further use. Due to skewed data, the Mann-Whitney test was used on these residuals to compare for group differences.

Pearson correlation coefficients between the CSF variables and the WML volumes were determined. To assess predictors for APP metabolites, scanner and sex were linearly regressed out from chronic WML and acute WML, again providing standardized residuals for further use. Consecutively, age, acute WML volume and chronic WML volume were entered as independent variables for all APP metabolite values. The different APP metabolites were sequentially entered as dependent variables in linear regression analysis.

## Results

**Figure 2:**
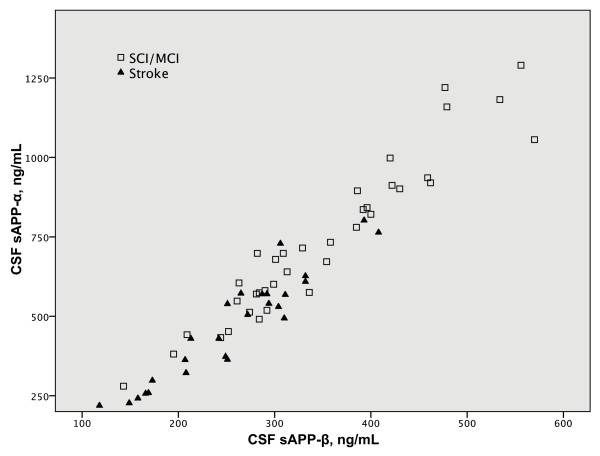
**Graph showing the correlation between the CSF levels of sAPP-α and sAPP-β in both groups of subjects**.

After regressing out age and sex from the biomarkers, the median CSF sAPP-α and sAPP-β levels were significantly lower (*p *< 0.001 for sAPP-α and *p *< 0.005 for sAPP-β) in the post-stroke patients than in the SCI/MCI patients. There were no significant differences in CSF levels of Aβ X-38, X-40 and X-42 between the two groups. The volume of chronic WML was higher in the stroke group than in the SCI/MCI group, but this difference was not significant (Table 2). The volume of acute post-stroke changes (infarct volume and surrounding WML halo) did not correlate with levels of CSF APP metabolites (data not shown). sAPP-α and sAPP-β levels were linearly related in both the stroke (*r *= 0.942, *p *< 0.001) and the SCI/MCI (*r *= 0.955, *p *< 0.001) groups (Fig 2).

**Table 2:** CSF concentrations of APP metabolites in two patient groups

Variable	Stroke median, (SD)	SCI/MCI median, (SD)	*p*
CSF sAPP-α ng/mL	499.5 (168.9)	698.0 (248.4)	<0.001

CSF sAPP-β ng/mL	258.0 (74.2)	329.0 (101.4)	<0.005

CSF Aβ X-38 pg/L	1048.0 (576.9)	1141.0 (810.1)	n.s.

CSF Aβ X-40 pg/L	5989.0 (1777.6)	6323.0 (2209.3)	n.s.

CSF AβX-42 pg/L	485.5 (167.5)	483.0 (227.7)	n.s.

Chronic WML cubic mm	5051.8 (11569.0)	971.0 (6105.8)	n.s.

Group differences after age and sex have been corrected for by linear regression. CSF sAPP-α and sAPP-β are significantly lower in the stroke group as compared to the SCI/MCI group. n.s.: not significant.

**Figure 3:**
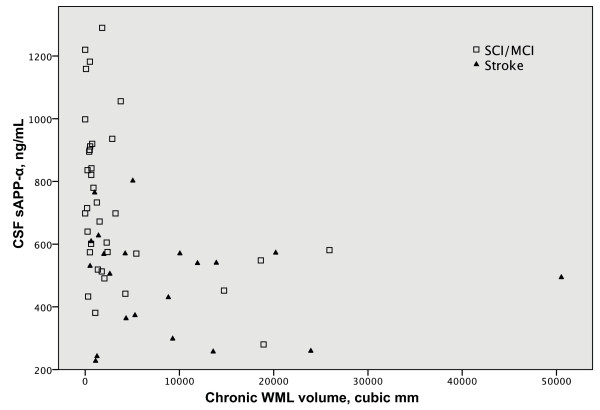
**Plot of CSF sAPP-α against chronic white matter lesion (WML) volume**. There were lower levels of APP metabolites in some patients with increased volumes of chronic WML.

Most patients with high chronic WML volumes had low CSF sAPP-α (Fig 3), and a clear negative correlation between the two variables was seen (r = -0.36, p < 0.01). A similar relation was seen between chronic WML volumes and CSF sAPP-β (r = -0.33, p < 0.05). In the regression analyses, chronic WML volume was a significant predictor for all examined CSF APP metabolites; *p *≤ 0.005 for sAPP-α, *p *≤ 0.01 for sAPP-β and AβX-38 levels, and *p *≤ 0.05 for AβX-40 and AβX-42 (Table 3). When repeating this analysis separately for the post-stroke and SCI/MCI groups, chronic WML still predicted sAPP-α and sAPP-β in the SCI/MCI group (*p *≤ 0.005 for both sAPP-α and sAPP-β), but not in the post-stroke group. Age was not significantly related to sAPP, but significantly predicted higher levels of AβX-38 (*p *≤ 0.01) and AβX-40 (*p *≤ 0.05). Sex and scanner site were not significantly related to any of the metabolites (data not shown).

**Table 3:** Chronic and acute white matter lesion volumes as predictors for APP metabolites in all patients.

Dependent Variables	Independent variables *p *(beta)		
	
	cWML	aWML	Age
CSF sAPP-α	≤0.005	n.s.	n.s.
	(-0.396)		

CSF sAPP-β	≤0.01	n.s.	n.s.
	(-0.369)		

CSF AβX-38	≤0.01	n.s.	≤0.01
	(-0.359)		(0.371)

CSF AβX-40	≤0.05	n.s.	≤0.05
	(-0.357)		(0.309)

CSF AβX-42	≤0.05	n.s.	n.s.
	(-0.273)		

Chronic WML significantly predicts CSF levels of APP metabolites.

cWML = chronic WML, aWML = acute WML.

## Discussion

We have shown that CSF levels of sAPP metabolites are lower in post-stroke patients than in SCI/MCI patients. There was an inverse relation between chronic, but not acute WML volume and all examined CSF APP metabolites across the groups. This suggests that the reduction is associated with the severity of chronic, but not acute ischemic disease.

One limitation of our study is that it did not include a healthy control group. It may therefore be argued that an alternative interpretation of our results would be that there was an increase of APP metabolite levels in the SCI/MCI group instead of a decrease in the post-stroke group. At least two pieces of evidence argue against this interpretation. Firstly, we did not see any significant difference in CSF levels of sAPP between controls and MCI patients in an earlier study from our laboratory [16]. Unfortunately however, the results from this study, although employing the same sAPP assay, cannot be directly compared to those in the present investigation due to batch-to-batch variation in sAPP concentration between different kits. Secondly, the inverse correlation of CSF sAPP levels with WML volume seen in the present study, as well as in an earlier investigation [29], suggest that subcortical changes are associated with sAPP reductions. Similar results have been reported in other diseases that are characterized by white matter changes, including multiple sclerosis [42] and dementia in acquired immunodeficiency syndrome [43]. It should be noted that Lewczuk and colleagues recently reported higher CSF concentrations of sAPP in patients with cognitive impairment (CI) *and *other neurochemical CSF findings characteristic of AD, than in patients with CI *without *CSF findings characteristic of AD [44]. Importantly, however, this study did not include a healthy control group and it is not unlikely that a significant number of the cognitively-impaired individuals without AD-like CSF biomarker changes in fact suffered from chronic cerebrovascular disease.

Our finding that increased chronic WML volume predicts lower sAPP metabolites is however clear-cut, and is most easily interpreted in favor of reduced sAPP metabolites in the stroke group. When repeating this analysis separately in the post-stroke and SCI/MCI groups, the prediction was not, however, significant in the stroke group, but was still significant within the SCI/MCI group. As CVD both increases APP production and impedes transport, this suggests that the net negative effects on APP levels occur at mild to moderate WML levels. Also, the stroke group was somewhat smaller than the SCI/MCI group, and this might explain why the prediction was not significant within the stroke group alone. In accord with earlier findings [16], sAPP-α and sAPP-β levels were very tightly correlated, indicating that the mechanism for the reduction lays upstream of α- and β-secretase activity. Energy-dependent fast axonal transport of APP [45] may well suffer in chronic ischemic brain disease affecting white matter tracts, resulting in reduced axonal transport of the precursor protein [34], reduced substrates for the secretases resulting in the observed reduction in metabolite levels in the CSF. Alternatively, ischemia may have an effect on earlier stages, e.g. reduce APP gene expression, but observations in experimental ischemia suggest that this is less likely [30,31].

Chronic WML volume predicts levels of both sAPP metabolites and Aβ peptides, but there is a significant difference between the post-stroke and the SCI/MCI groups for sAPP, suggesting that for the amyloid β peptides there is more extensive interplay with other factors. In a previous study [46], acute stroke was not shown to have a significant short term effect on levels of AβX-42. However, as human Aβ clearance rates are close to 10% per hour [47]; changes temporally related to acute stroke onset are not expected to be detected in the present study.

## Conclusions

In the patient group as a whole, there was a strong correlation between CSF sAPP-α and sAPP-β concentrations. An inverse relationship was demonstrated between the volume of chronic WML and CSF APP metabolites (sAPP-α, sAPP-β, Aβ X-38, X-40 and X-42) in both stroke patients and SCI/MCI patients. In addition, there were lower levels of CSF sAPP-α and sAPP-β in the stroke group when compared to the SCI/MCI-group. This suggests that ischemia influences APP metabolism probably through inhibition of fast axonal transport of APP. If confirmed, the present results implicate new mechanisms for reduction of CSF APP metabolites, including CSF AβX-42, which is a predictor for development of AD.

## Competing interests

The authors declare that they have no competing interests.

## Authors' contributions

PS determined the WML volumes and performed the statistical analyses, participated in the collection of data and in drafting of the manuscript. KB participated in the immunological analyses and the conception of the study. HZ participated in the immunological analyses and the conception of the study. RG participated in the collection of data. LR participated in the immunological analyses and the conception of the study. LJ participated in the collection of data and biosamples. VS participated in the collection of data. TF participated in the conception of the study, its design and coordination and helped to draft the manuscript. All authors read and approved the final manuscript.

## References

[B1] Selkoe DJ Podlisny MB Deciphering the genetic basis of Alzheimer's disease Annu Rev Genomics Hum Genet 2002 3 67 99 10.1146/annurev.genom.3.022502.103022 12142353

[B2] Chai CK The genetics of Alzheimer's disease Am J Alzheimers Dis Other Demen 2007 22 37 41 10.1177/1533317506295655 17534000 PMC10697204

[B3] Blennow K de Leon MJ Zetterberg H Alzheimer's disease Lancet 2006 368 387 403 10.1016/S0140-6736(06)69113-7 16876668

[B4] Braak H Braak E Evolution of the neuropathology of Alzheimer's disease Acta Neurol Scand Suppl 1996 165 3 12 8740983 10.1111/j.1600-0404.1996.tb05866.x

[B5] Fagan AM Mintun MA Mach RH Lee SY Dence CS Shah AR LaRossa GN Spinner ML Klunk WE Mathis CA DeKosky ST Morris JC Holtzman DM Inverse relation between in vivo amyloid imaging load and cerebrospinal fluid Abeta42 in humans Ann Neurol 2006 59 512 519 10.1002/ana.20730 16372280

[B6] Koo EH Sisodia SS Archer DR Martin LJ Weidemann A Beyreuther K Fischer P Masters CL Price DL Precursor of amyloid protein in Alzheimer disease undergoes fast anterograde axonal transport Proc Natl Acad Sci USA 1990 87 1561 1565 10.1073/pnas.87.4.1561 1689489 PMC53515

[B7] Portelius E Zetterberg H Gobom J Andreasson U Blennow K Targeted proteomics in Alzheimer's disease: focus on amyloid-beta Expert Rev Proteomics 2008 5 225 237 10.1586/14789450.5.2.225 18466053

[B8] Czirr E Cottrell BA Leuchtenberger S Kukar T Ladd TB Esselmann H Paul S Schubenel R Torpey JW Pietrzik CU Golde TE Wiltfang J Baumann K Koo EH Weggen S Independent generation of Abeta42 and Abeta38 peptide species by gamma-secretase J Biol Chem 2008 283 17049 17054 10.1074/jbc.M802912200 18426795

[B9] Findeis MA The role of amyloid beta peptide 42 in Alzheimer's disease Pharmacol Ther 2007 116 2 266 86 Epub 2007 Jul 17 10.1016/j.pharmthera.2007.06.006 17716740

[B10] Strozyk D Blennow K White LR Launer LJ CSF Abeta 42 levels correlate with amyloid-neuropathology in a population-based autopsy study Neurology 2003 60 652 656 12601108 10.1212/01.wnl.0000046581.81650.d0

[B11] Hansson O Zetterberg H Buchhave P Londos E Blennow K Minthon L Association between CSF biomarkers and incipient Alzheimer's disease in patients with mild cognitive impairment: a follow-up study Lancet Neurol 2006 5 228 234 10.1016/S1474-4422(06)70355-6 16488378

[B12] Young-Pearse TL Chen AC Chang R Marquez C Selkoe DJ Secreted APP regulates the function of full-length APP in neurite outgrowth through interaction with integrin beta1 Neural Develop 2008 3 15 10.1186/1749-8104-3-15 PMC2442059 18573216

[B13] Taylor CJ Ireland DR Ballagh I Bourne K Marechal NM Turner PR Bilkey DK Tate WP Abraham WC Endogenous secreted amyloid precursor protein-alpha regulates hippocampal NMDA receptor function, long-term potentiation and spatial memory Neurobiol Dis 2008 31 250 260 10.1016/j.nbd.2008.04.011 18585048

[B14] Turner PR O'Connor K Tate WP Abraham WC Roles of amyloid precursor protein and its fragments in regulating neural activity, plasticity and memory Prog Neurobiol 2003 70 1 32 10.1016/S0301-0082(03)00089-3 12927332

[B15] Gauthier S Reisberg B Zaudig M Petersen RC Ritchie K Broich K Belleville S Brodaty H Bennett D Chertkow H Cummings JL de Leon M Feldman H Ganguli M Hampel H Scheltens P Tierney MC Whitehouse P Winblad B Mild cognitive impairment Lancet 2006 367 1262 1270 10.1016/S0140-6736(06)68542-5 16631882

[B16] Zetterberg H Andreasson U Hansson O Wu G Sankaranarayanan S Andersson ME Buchhave P Londos E Umek RM Minthon L Simon AJ Blennow K Elevated cerebrospinal fluid BACE1 activity in incipient Alzheimer disease Arch Neurol 2008 65 1102 1107 10.1001/archneur.65.8.1102 18695061

[B17] Reisberg B Prichep L Mosconi L John ER Glodzik-Sobanska L Boksay I Monteiro I Torossian C Vedvyas A Ashraf N Jamil IA de Leon MJ The pre-mild cognitive impairment, subjective cognitive impairment stage of Alzheimer's disease Alzheimers Dement 2008 4 S98 S108 10.1016/j.jalz.2007.11.017 18632010

[B18] Reisberg B Gauthier S Current evidence for subjective cognitive impairment (SCI) as the pre-mild cognitive impairment (MCI) stage of subsequently manifest Alzheimer's disease Int Psychogeriatr 2008 20 1 16 18072981 10.1017/S1041610207006412

[B19] Kuller LH Lopez OL Newman A Beauchamp NJ Burke G Dulberg C Fitzpatrick A Fried L Haan MN Risk factors for dementia in the cardiovascular health cognition study Neuroepidemiology 2003 22 13 22 10.1159/000067109 12566949

[B20] Snowdon DA Greiner LH Mortimer JA Riley KP Greiner PA Markesbery WR Brain infarction and the clinical expression of Alzheimer disease. The Nun Study Jama 1997 277 813 817 10.1001/jama.277.10.813 9052711

[B21] Vermeer SE Prins ND den Heijer T Hofman A Koudstaal PJ Breteler MM Silent brain infarcts and the risk of dementia and cognitive decline N Engl J Med 2003 348 1215 1222 10.1056/NEJMoa022066 12660385

[B22] Schmidt R Scheltens P Erkinjuntti T Pantoni L Markus HS Wallin A Barkhof F Fazekas F White matter lesion progression: a surrogate endpoint for trials in cerebral small-vessel disease Neurology 2004 63 139 144 15249623 10.1212/01.wnl.0000132635.75819.e5

[B23] Inzitari D Pracucci G Poggesi A Carlucci G Barkhof F Chabriat H Erkinjuntti T Fazekas F Ferro JM Hennerici M Langhorne P O'Brien J Scheltens P Visser MC Wahlund LO Waldemar G Wallin A Pantoni L Changes in white matter as determinant of global functional decline in older independent outpatients: three year follow-up of LADIS (leukoaraiosis and disability) study cohort BMJ 2009 339 b2477 10.1136/bmj.b2477 19581317 PMC2714680

[B24] Pantoni L Garcia JH The significance of cerebral white matter abnormalities 100 years after Binswanger's report. A review Stroke 1995 26 1293 1301 7604429 10.1161/01.str.26.7.1293

[B25] Bigler ED Kerr B Victoroff J Tate DF Breitner JC White matter lesions, quantitative magnetic resonance imaging, and dementia Alzheimer Dis Assoc Disord 2002 16 161 170 10.1097/00002093-200207000-00006 12218647

[B26] Formichi P Parnetti L Radi E Cevenini G Dotti MT Federico A CSF levels of beta-amyloid 1-42, tau and phosphorylated tau protein in CADASIL Eur J Neurol 2008 15 1252 1255 10.1111/j.1468-1331.2008.02277.x 18803653

[B27] Stefani A Bernardini S Panella M Pierantozzi M Nuccetelli M Koch G Urbani A Giordano A Martorana A Orlacchio A Federici G Bernardi G AD with subcortical white matter lesions and vascular dementia: CSF markers for differential diagnosis J Neurol Sci 2005 237 83 88 10.1016/j.jns.2005.05.016 15990115

[B28] Stenset V Johnsen L Kocot D Negaard A Skinningsrud A Gulbrandsen P Wallin A Fladby T Associations between white matter lesions, cerebrovascular risk factors, and low CSF Abeta42 Neurology 2006 67 830 833 10.1212/01.wnl.0000234030.77831.5a 16966546

[B29] Jonsson M Zetterberg H van Straaten E Lind K Syversen S Edman A Blennow K Rosengren L Pantoni L Inzitari D Wallin A Cerebrospinal fluid biomarkers of white matter lesions - cross-sectional results from the LADIS study Eur J Neurol 2009 17 3 377 82 Epub 2009 Oct 21 10.1111/j.1468-1331.2009.02808.x 19845747

[B30] Badan I Dinca I Buchhold B Suofu Y Walker L Gratz M Platt D Kessler CH Popa-Wagner A Accelerated accumulation of N- and C-terminal beta APP fragments and delayed recovery of microtubule-associated protein 1B expression following stroke in aged rats Eur J Neurosci 2004 19 2270 2280 10.1111/j.0953-816X.2004.03323.x 15090053

[B31] Yam PS Takasago T Dewar D Graham DI McCulloch J Amyloid precursor protein accumulates in white matter at the margin of a focal ischaemic lesion Brain Res 1997 760 150 157 10.1016/S0006-8993(97)00290-4 9237529

[B32] Wen Y Onyewuchi O Yang S Liu R Simpkins JW Increased beta-secretase activity and expression in rats following transient cerebral ischemia Brain Res 2004 1009 1 8 10.1016/j.brainres.2003.09.086 15120577

[B33] Shah SB Nolan R Davis E Stokin GB Niesman I Canto I Glabe C Goldstein LS Examination of potential mechanisms of amyloid-induced defects in neuronal transport Neurobiol Dis 2009 36 11 25 10.1016/j.nbd.2009.05.016 19497367

[B34] Suenaga T Ohnishi K Nishimura M Nakamura S Akiguchi I Kimura J Bundles of amyloid precursor protein-immunoreactive axons in human cerebrovascular white matter lesions Acta Neuropathol 1994 87 450 455 10.1007/BF00294171 8059597

[B35] Gotz J Ittner LM Kins S Do axonal defects in tau and amyloid precursor protein transgenic animals model axonopathy in Alzheimer's disease? J Neurochem 2006 98 993 1006 10.1111/j.1471-4159.2006.03955.x 16787410

[B36] Reisberg B Ferris SH de Leon M Crook T Global Deterioration Scale (GDS) Psychopharmacol Bull 1988 24 661 663 3249768

[B37] Morris JC Clinical dementia rating: a reliable and valid diagnostic and staging measure for dementia of the Alzheimer type Int Psychogeriatr 1997 9 Suppl 1 173 176 discussion 177-178 10.1017/S1041610297004870 9447441

[B38] Folstein MF Folstein SE McHugh PR "Mini-mental state". A practical method for grading the cognitive state of patients for the clinician J Psychiatr Res 1975 12 189 198 10.1016/0022-3956(75)90026-6 1202204

[B39] Wallin A Edman A Blennow K Gottfries CG Karlsson I Regland B Sjogren M Stepwise comparative status analysis (STEP): a tool for identification of regional brain syndromes in dementia J Geriatr Psychiatry Neurol 1996 9 185 199 8970012 10.1177/089198879600900406

[B40] Royall DR Mahurin RK Gray KF Bedside assessment of executive cognitive impairment: the executive interview J Am Geriatr Soc 1992 40 1221 1226 1447438 10.1111/j.1532-5415.1992.tb03646.x

[B41] Kiernan RJ Mueller J Langston JW van Dyke C The Neurobehavioral Cognitive Status Examination: A Brief But Differentiated Approach to Cognitive Assessment Ann Intern Med 1987 450 481 485 107PBS Record 10.7326/0003-4819-107-4-481 3631786

[B42] Mattsson N Axelsson M Haghighi S Malmestrom C Wu G Anckarsater R Sankaranarayanan S Andreasson U Fredrikson S Gundersen A Johnsen L Fladby T Tarkowski A Trysberg E Wallin A Anckarsater H Lycke J Andersen O Simon AJ Blennow K Zetterberg H Reduced cerebrospinal fluid BACE1 activity in multiple sclerosis Mult Scler 2009 15 448 454 10.1177/1352458508100031 19153172

[B43] Gisslen M Krut J Andreasson U Blennow K Cinque P Brew BJ Spudich S Hagberg L Rosengren L Price RW Zetterberg H Amyloid and tau cerebrospinal fluid biomarkers in HIV infection BMC Neurol 2009 9 63 10.1186/1471-2377-9-63 20028512 PMC2807422

[B44] Lewczuk P Kamrowski-Kruck H Peters O Heuser I Jessen F Popp J Burger K Hampel H Frolich L Wolf S Prinz B Jahn H Luckhaus C Perneczky R Hull M Schroder J Kessler H Pantel J Gertz HJ Klafki HW Kolsch H Reulbach U Esselmann H Maler JM Bibl M Kornhuber J Wiltfang J Soluble amyloid precursor proteins in the cerebrospinal fluid as novel potential biomarkers of Alzheimer's disease: a multicenter study Mol Psychiatry 15 138 145 10.1038/mp.2008.84 18663368

[B45] Kins S Lauther N Szodorai A Beyreuther K Subcellular trafficking of the amyloid precursor protein gene family and its pathogenic role in Alzheimer's disease Neurodegener Dis 2006 3 218 226 10.1159/000095259 17047360

[B46] Hesse C Rosengren L Vanmechelen E Vanderstichele H Jensen C Davidsson P Blennow K Cerebrospinal fluid markers for Alzheimer's disease evaluated after acute ischemic stroke J Alzheimers Dis 2000 2 199 206 12214084 10.3233/jad-2000-23-402

[B47] Bateman RJ Munsell LY Morris JC Swarm R Yarasheski KE Holtzman DM Human amyloid-beta synthesis and clearance rates as measured in cerebrospinal fluid in vivo Nat Med 2006 12 856 861 10.1038/nm1438 16799555 PMC2983090

